# Using approximate Bayesian inference for a “steps and turns” continuous-time random walk observed at regular time intervals

**DOI:** 10.7717/peerj.8452

**Published:** 2020-02-11

**Authors:** Sofia Ruiz-Suarez, Vianey Leos-Barajas, Ignacio Alvarez-Castro, Juan Manuel Morales

**Affiliations:** 1 INIBIOMA (CONICET-Universidad Nacional del Comahue), Rio Negro, Argentina; 2 Facultad de Ciencias Económicas, Universidad Nacional de Rosario, Rosario, Argentina; 3 Department of Statistics, North Carolina State University, Raleigh, United States of America; 4 Department of Forestry and Environmental Resources, North Carolina State University, Raleigh, NC, United States of America; 5 Universidad de la República, Montevideo, Uruguay

**Keywords:** Movement Ecology, Approximate Bayesian Computation, Observation Time-Scale, Random walk, Simulated Trajectories, Animal Movement

## Abstract

The study of animal movement is challenging because movement is a process modulated by many factors acting at different spatial and temporal scales. In order to describe and analyse animal movement, several models have been proposed which differ primarily in the temporal conceptualization, namely continuous and discrete time formulations. Naturally, animal movement occurs in continuous time but we tend to observe it at fixed time intervals. To account for the temporal mismatch between observations and movement decisions, we used a state-space model where movement decisions (steps and turns) are made in continuous time. That is, at any time there is a non-zero probability of making a change in movement direction. The movement process is then observed at regular time intervals. As the likelihood function of this state-space model turned out to be intractable yet simulating data is straightforward, we conduct inference using different variations of Approximate Bayesian Computation (ABC). We explore the applicability of this approach as a function of the discrepancy between the temporal scale of the observations and that of the movement process in a simulation study. Simulation results suggest that the model parameters can be recovered if the observation time scale is moderately close to the average time between changes in movement direction. Good estimates were obtained when the scale of observation was up to five times that of the scale of changes in direction. We demonstrate the application of this model to a trajectory of a sheep that was reconstructed in high resolution using information from magnetometer and GPS devices. The state-space model used here allowed us to connect the scales of the observations and movement decisions in an intuitive and easy to interpret way. Our findings underscore the idea that the time scale at which animal movement decisions are made needs to be considered when designing data collection protocols. In principle, ABC methods allow to make inferences about movement processes defined in continuous time but in terms of easily interpreted steps and turns.

## Introduction

The way in which animals move is of fundamental importance in ecology and evolution. It plays important roles in the fitness of individuals and in gene exchange (Nathan et al., 2008), in the structuring of populations and communities (Turchin, 1998; Matthiopoulos et al., 2015; Morales et al., 2010), and in the spread of diseases (Fvre et al., 2006). The study of movement is challenging because it is a process modulated by many factors acting at different spatial and temporal scales  (Gurarie & Ovaskainen, 2011; Hooten et al., 2017). In general, the process of animal movement occurs in continuous time but we observe individual locations at time intervals dictated by logistical constrains such as battery life. When modeling trajectories, it is common to assume that the time scale at which animals decide to change their movement is the same as that of the observation. However, it is not always in that way, and it is necessary to be aware of this fact in order to avoid drawing conclusions about the movement process that depend on the time scale at which the observations were taken.

In this context, state-space models provide a convenient tool for movement data analysis (Patterson et al., 2008). The main idea is to estimate the latent movement process or characteristics of it given the observational process. Thus, they consist of two stochastic models: a latent (or state) model and an observation model. The first describes the state of the animal (it could be the location, behaviour, etc.) and the second one describes the observation of the state, possibly with some measurement error. In the case of this study, the aim is to estimate the parameters that governs the state process given the information from the observations.

Several state-space models have been proposed for animal movement differing primarily in the temporal conceptualization of the movement process, namely discrete and continuous time formulations (McClintock et al., 2014). On the one hand, discrete-time models describe movement as a series of steps and turns (or movement directions) that are performed at regular occasions (Morales et al., 2004; Jonsen, Flemming & Myers, 2005; McClintock et al., 2012). The advantage of this approach is that it allows the dynamics involved in the movement process to be conceptualized in a simple and intuitive way, which facilitates implementation and interpretation. Typically, in these models the temporal scales of both the state and the observation process are the same. Thus, the observation times coincide with the times in which the animal are assumed to make movement decisions. On the other hand, continuous-time models have been proposed (Blackwell, 1999; Jonsen, Flemming & Myers, 2005; Harris & Blackwell, 2013) in which the movement process is defined for any time and are usually expressed through stochastic differential equations that account for the dependence between successive locations. Consequently, they are not dependent on a particular timescale and, within reasonable limits, a continuous-time analysis yields the same results regardless of the temporal resolution of observations.

The continuous-time approach has the advantage of being more realistic and that it avoids dependence on a particular timescale. A possible drawback is in the interpretation of instantaneous movement parameters (e.g., those related to Ornstein–Uhlenbeck processes and other diffusion models). However, as it is mentioned in McClintock et al. (2014), for any continuous- or discrete-time approach to be useful, the temporal resolution of the observed data must be relevant to the specific movement behaviors of interest. While both approaches have advantages and disadvantages, the discrete-time models are more intuitive and easy to interpret yet can be considered less realistic than continuous time models  (McClintock et al., 2014).

We present a state-space model that formulates the movement process in continuous time and the observation in discrete time (regular intervals). Although the formulation is based on steps and turns, the probability of a change in the trajectory is positive for all times *t*, and thus the movement process is continuous over time. In most cases, when trying to make inferences about movement processes, only location data observed at regular times is available. For example, it is common to obtain GPS fixes at time intervals that are dictated by logistics constraints such as battery life. Therefore, the number and times at which there were changes in movement between successive recorded locations is information that is not available and that it is usually ignored. It is common to assume that the time scale at which animals make their movement decisions is the same as the time scale at which the location data was taken. However, this is not always the case and, under certain circumstances, this assumption may lead to incorrect inferences and interpretations of the movement process.

Our goals here are to combine the ease of interpretation of models based on steps and turns with the realism of continuous-time models, and to analyze the relationship between the scales of observations and movement decisions. We use a random walk where the movement decisions (steps and turns) can be made at any point in time while the movement process is observed at regular time intervals. In this model there are two different time scales: one for the state process and one for the observation. The advantage here is that this model allows us to differentiate between the times in which the animals make movement decisions and the times in which the observations are made.

We then assessed the capacity of Approximate Bayesian Computation (ABC) methods recovering the parameters that govern the state process as a function of these two time scales.

Parton & Blackwell (2017) present a continuous time movement model which has some similarities to the model we present here. Parton and Blackwell’s model is formulated using stochastic differential equations in terms of bearings and speed. The animal’s locations are then observed at discrete times with certain error. Both models are similar as they assume that changes in the direction of the trajectory can be at any time, that the trajectory is correlated, and that observations are at discrete times. However, the formulations are different as in Parton and Blackwell’s model speed is stochastic and there is an observation error. They notice that the likelihood is intractable due to the complicated relationship between the locations and parameters when the changes in bearing and speed are unobserved. For inference they develop a Markov Chain Monte Carlo algorithm which involves augmenting observed locations with a reconstruction of the underlying movement process.

As in Parton and Blackwell’s model, our proposed model formulation has an intractable likelihood. However, simulating the movement and its observation is straightforward, suggesting that likelihood-free methods such as Approximate Bayesian Computation could be useful (Beaumont, 2010; Csilléry et al., 2010). These techniques have been used to fit models that involve movement processes such as overall rate of movement (Van der Vaart et al., 2015), or movement among patches in individual based models of meta-population dynamics (Sirén et al., 2018). However, as far as we are aware, they have not been used to make inferences about movement trajectories.

Here we describe, formalize, and expose the possible complications of a state-space movement model with two different temporal scales. We use stochastic simulations to evaluate the ability of three ABC techniques to recover the parameter values driving the movement process. Keeping in mind the ecological purpose behind implementing such a model, we assess the quality of these estimations with regard to the relationship between the two temporal scales. Finally, we apply the model to a high resolution trajectory of sheep to evaluate the performance of the ABC inference with real data.

## Methods

### Movement model with random time between movement decisions

Many animal movement studies assume discrete time correlated random walks (CRW) as building blocks to more complex models (Turchin, 1998; Morales et al., 2004). At every time step, an individual chooses a turning angle (difference between the previous and new movement direction) and a speed. The turning angle distribution is concentrated around zero, resulting in short-term correlations in the direction of movement (persistence in movement direction). Here, we allow changes in direction to occur at any point in time. An individual moves in a certain direction for a certain period of time, and then it makes a turn and starts moving in a new direction for another period of time. Our movement model is a form of the velocity jump process in which the motion consists of a sequence of ”runs” separated by reorientations, during which a new velocity is chosen (Othmer, Dunbar & Alt, 1988; Codling, Plank & Benhamou, 2008). In our model, the speed of movement during the active phase is constant, and the temporal scale of the waiting time of the reorientation phase is considered instantaneous.

Since, in practice, the path of an animal is usually observed at particular sampling occasions, we consider that the observation occurs at regular time intervals. Therefore the observation lies in the location of the individual every time Δ*t*. As a simplification, we assume that there is no observation error.

Assuming constant movement speed, let the variable *M*_*i*_ describes the position of the state process at step *i*, presented in *x*–*y* coordinates, i.e.,  *M*_*i*_ = (*m*_*i*,1_, *m*_*i*,2_) where *i* represents an index of the time over the steps for *i* = 0, …, *N*_*steps*_. Given, *m*_0,1_ = 0 and *m*_0,2_ = 0, we have for *i* = 1, …, *N*_*steps*_ that,

(1) \begin{eqnarray*}\begin{array}{@{}cc@{}} \displaystyle {m}_{i,1}=&\displaystyle {m}_{i-1,1}+cos({\phi }_{i-1}){t}_{i-1}\\ \displaystyle {m}_{i,2}=&\displaystyle {m}_{i-1,2}+sin({\phi }_{i-1}){t}_{i-1}\\ \displaystyle {\phi }_{i}=&\displaystyle \sum _{k=1}^{i}{\omega }_{i} \end{array}\end{eqnarray*}

where *t*_*i*_ is the duration of step *i*, and *ω*_*i*_ is the turning angle between steps *i* and *i* + 1, so that *ϕ*_*i*_ represents the direction of the step *i*. Each *t*_*i*_ is assumed to be independently drawn from an exponential distribution with parameter *λ* and each *ω*_*i*_ from a von Mises distribution with a fixed mean *ν* = 0 and parameter *κ* for the concentration  (Wu et al., 2000; Codling, Plank & Benhamou, 2008). While the model can be extended to allow *κ* and *λ* to depend on the landscape, environment, or animal behaviour, for this work we only consider the initial case as an starting point.

Next, we define the observation and its links with the state movement process (Eq. (2)). Let *O*_*j*_ = (*o*_*j*,1_, *o*_*j*,2_) denote the position of observation *j* in *x*–*y* coordinates, with *j* = 0, …, *N*_*obs*_. A second index *j* is used for the time over the observations. We defined *T*_*i*_ as the time in which the change of direction *i* took place:

\begin{eqnarray*}\begin{array}{@{}l@{}} \displaystyle {T}_{0}=0\\ \displaystyle {T}_{i}=\sum _{k=0}^{i-1}{t}_{k} \text{for} i=1,\ldots ,{N}_{steps}. \end{array} \end{eqnarray*}

In addition it is necessary to determine the number of changes in direction that occurred before a given observation, we define *N*_*j*_ as the number of steps (or changes in direction) that the animal took from time 1 to time *j*Δ*t*.

*O*_0_ = *M*_0_

And for *j* = 1, …, *N*_*obs*_

(2) \begin{eqnarray*}\begin{array}{@{}c@{}} \displaystyle {o}_{j,1}={m}_{{N}_{j},1}+cos({\phi }_{{N}_{j}}) \left( j\Delta t-{T}_{{N}_{j}} \right) \\ \displaystyle {o}_{j,2}={m}_{{N}_{j},2}+sin({\phi }_{{N}_{j}}) \left( j\Delta t-{T}_{{N}_{j}} \right) . \end{array}\end{eqnarray*}

**Figure 1:**
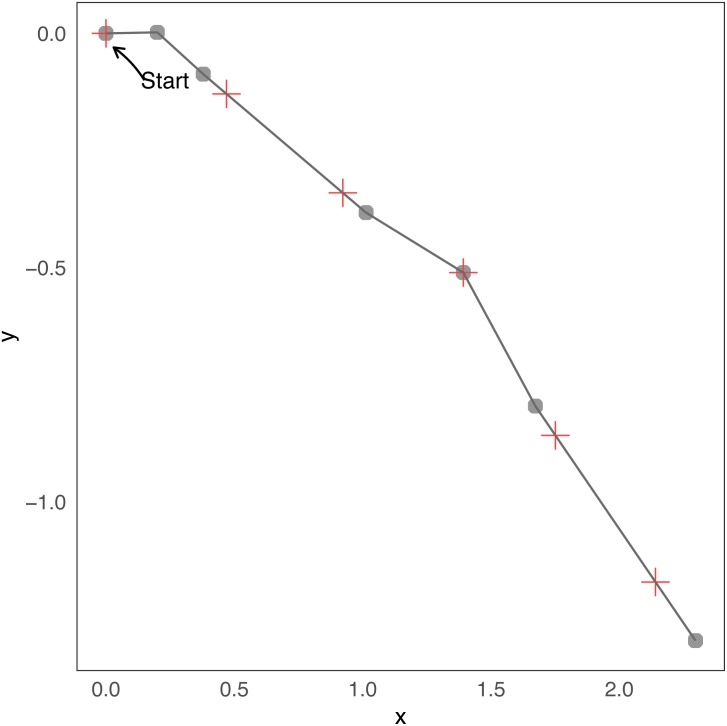
Example of few steps. Minimal example of a simulation from the continuous-time “steps and turns” model. Changes in direction are indicated with gray dots, and observations with red crosses. The trajectory starts at coordinates (0, 0). Before the first and the second red crosses (the initial one does not count, *i* = 0) there are two grey points, then the number of changes before observation 1 and 2 are *N*_1_ = *N*_2_ = 2. Before the third red cross there are four grey points, then *N*_3_ = 4, etc.

Note that *N*_*j*_ is the index that corresponds to the maximum time *T*_*i*_ less or equal to *j*Δ*t*, i.e., ${N}_{j}=\max \left\{ m/{T}_{m}\leq j\Delta t \right\} $. Therefore, the location *j* is the last location of the state process given by *N*_*j*_ plus the difference between *j*Δ*t* and the time at which the step *N*_*j*_ was produced in the direction *ϕ*_*N*_*j*__. To better understand this relationship consider a minimal example of a few steps. Assuming the duration of steps and turning angles of Table 1 and Δt=0 .5, in Fig. 1 we present a short trajectory showing the locations of changes in direction and observations at regular times. In that case *N*_1_ = 2, because *T*_1_ = *t*_0_ = 0.2 ≤ 1Δ*t*, *T*_2_ = *t*_0_ + *t*_1_ = 0.4 ≤ 1Δ*t* but *T*_3_ = *t*_0_ + *t*_1_ + *t*_2_ = 1.1⁄ ≤ 1Δ*t*. With the same reasoning *N*_2_ = 2, *N*_3_ = 4, *N*_4_ = 5, *N*_5_ = 5, etc.

**Table 1:** Values for the turning angles and duration of steps for the example of Fig. 1.

Duration of steps (${t}_{i}^{{^{\prime}}}s$)	0.2	0.2	0.7	0.4	0.4	0.8
Turning angle (${\omega }_{i}^{{^{\prime}}}s$)	0.32	5.65	5.81	0.02	0.11	5.81

### Expression for the likelihood function

In order to construct the likelihood we first present the *complete-data* likelihood, i.e., we assume that we know all the values for *t*_*i*_ and *ω*_*i*_. In that case, as *O*|*M* is deterministic, we have

\begin{eqnarray*}L(\kappa ,\lambda ;M,O)& =P(O,M{|}\kappa ,\lambda )\nonumber\\\displaystyle & =P(O{|}M,\kappa ,\lambda )P(M{|}\kappa ,\lambda )\nonumber\\\displaystyle & =P(M{|}\kappa ,\lambda )\nonumber\\\displaystyle & =P({t}_{1},...,{t}_{{N}_{steps}}{|}\lambda )P({\omega }_{1},...,{\omega }_{{N}_{steps}}{|}\kappa ) \end{eqnarray*}

Suppose that we do not know the values of *t*_*i*_ and *ω*_*i*_, but do know the number of steps that the animal took between consecutive observations, *N*_*j*_∀*j*. In that case, it would be necessary to obtain the distributions of *M*_*i*_ (Eq. (1)) (or at least of a proportion of them), which are not available in closed form.

Finally, to formulate the marginal likelihood, *L*(*κ*, *λ*), it is further necessary to integrate over all the possible values of *N*_*j*_, by determining *P*(*N*_*j*_ = *r*) for *r* ∈ ℕ, which can be, in principle, infinite. Obtaining the expression for and evaluation of the likelihood results to be a complex task. Likelihood-free methods that allow one to circumvent the need to evaluate the likelihood, such as ABC, have proven to be useful in these cases.

###  Inference using Approximate Bayesian Computation

Approximate Bayesian Computation (ABC) is a family of simulation-based techniques to obtain posterior samples in models with an intractable likelihood function. In recent years, ABC has become popular in a diverse range of fields (Sisson, Fan & Beaumont, 2018) such as molecular genetics (Marjoram & Tavar, 2006), epidemiology (Tanaka et al., 2006; McKinley, Cook & Deardon, 2009; Lopes & Beaumont, 2010), evolutionary biology (Bertorelle, Benazzo & Mona, 2010; Csilléry et al., 2010; Baudet et al., 2015), and ecology (Beaumont, 2010; Sirén et al., 2018). This approach is also useful when the computational effort to calculate the likelihood is large compared to that of the simulation of the model of interest. The likelihood function described earlier turns out to be complex to calculate, yet it is easy to simulate trajectories from the statistical model, based on independent draws from exponential and von Mises distributions combined with the observations at regular time intervals.

Let *θ* denote the vector of parameter of interest and ***y*** denote the observed data. The posterior distribution *p*(*θ*∣***y***) is proportional to the product of the prior distribution *p*(*θ*) and the likelihood function *p*(***y***∣*θ*)

*p*(*θ*∣***y***) ∝ *p*(*θ*)*p*(***y***∣*θ*)

The basic idea of ABC methods is to obtain simulations from the joint distribution, *p*(***y***, *θ*) and retain the parameter values that generate simulated data close to the observed data, (*y*). In this way, ABC methods aim to replace the likelihood function with a measure of similarity between simulated data and actual data.

The rejection algorithm is the simplest and first proposed method to perform ABC (Tavaré et al., 1997; Pritchard et al., 1999). It can be described as follows:

1. Compute a vector of summary statistics with observed data, *S*(***y***).
2. Simulate parameters *θ*_∗_ sampled from *p*(*θ*) and data ***y***_∗_ sampled from *p*(.∣*θ*_∗_).
3. Compute a vector of summary statistics with simulated data, *S*(***y***_∗_).
4. *θ*_∗_ is accepted as a posterior sample, if *ρ*(*S*(***y***_∗_), *S*(***y***)) < *δ*, for some distance measure *ρ* and threshold *δ*.
5. Repeat 2–4 ***K*** times.

The above rejection algorithm produces samples from *p*(*θ*∣*ρ*(*S*(***y***), *S*(***y***_∗_))) < *δ* which is an approximation of *p*(*θ*∣***y***). In particular, when the summary statistics are sufficient or near-sufficient for *ρ*, the approximate posterior distribution converges to the true posterior distribution as *δ* goes to 0 (Marjoram et al., 2003). Instead of selecting a value for *δ*, it is a common practice to set a threshold *ϵ* as a tolerance level to define the proportion of accepted simulations. For a complete review of ABC methods and techniques see (Csilléry et al., 2010; Beaumont, 2010; Sisson, Fan & Beaumont, 2018).

We consider two regression-based correction methods. These implement an additional step to correct the imperfect match between the accepted and observed summary statistics. One of these use local linear regression (Beaumont, Zhang & Balding, 2002), and the other is based on neural networks (Blum & François, 2010). To make the correction, both methods use the regression equation given by

*θ*_*i*_ = *r*(*S*(*y*_*i*_)) + *ξ*_*i*_

where *r* is the regression function and the *ξ*_*i*_’s are centered random variables with equal variance. For the linear correction *r* is assumed to be linear function and for the neural network correction *r* is not necessary linear. A weight *K*[*d*(*S*(*y*_*i*_), *S*(*y*_0_))] (for *K* a statistical kernel) is assigned to each simulation, so those closer to the observed summary statistics are given greater weight. The *r* and *ξ* values can be estimated by fitting a linear regression in the first case and a feed-forward neural network regression in the second case. Then, a weighted sample from the posterior distribution is obtained by considering ${\theta }_{i}^{corr}$ as follows ${\theta }_{i}^{corr}=\hat {r}(S({y}_{0}))+{\hat {\xi }}_{i}$

where $\hat {r}(.)$ is the estimated conditional mean and the ${\hat {\xi }}_{i}$s are the empirical residuals of the regression.

After a preliminary analysis, in which 20 summary statistics were assessed, we choose four that characterize the trajectories according to parameter values. Looking for summaries that capture diverse features of the movement, we plotted the proposed summaries against known parameters and decided to keep those summary statistics that changed monotonically with parameters values. The plots of all the summaries assessed are provided in the Appendix S1. Finally, the four selected summaries were: the inverse of the observed average step length (where an observed step is the distance between positions of consecutive observed times); a point estimator for *κ*, calculated as the inverse function of the ratio of the first and zero order Bessel functions of the first kind evaluated at the mean of the cosine of the observed turning angles (where observed turning angles were determined as the difference between consecutive directions in the observations); the standard deviation of the observed turning angles and lastly, the standard deviation of the observed step lengths (Table 2).

**Table 2:** Summary statistics selected to fit the model using the three ABC algorithms.

**Summary statistic**	**Formula**
(1)The inverse of the observed average step	$1/{\mathop{\sum }\nolimits }_{j=0}^{{N}_{obs}}\sqrt{({o}_{j+1,1}-{o}_{j,1})^{2}+({o}_{j+1,2}-{o}_{j,2})^{2}}$,
(2) Point estimate for *κ*	${A}^{-1} \left( \frac{{\mathop{\sum }\nolimits }_{j=1}^{{N}_{obs}}\cos ({\omega }_{obs,j})}{{N}_{obs}} \right) $ Where ${\omega }_{obs,j}=\arctan ( \frac{{o}_{j+1,1}-{o}_{j,1}}{{o}_{j+1,2}-{o}_{j,2}} )$ and $A(x)= \frac{{I}_{1}(x)}{{I}_{0}(x)} $Hornik & Grün (2014)
(3) Standard deviation of the turning angle	$\sqrt{ \frac{{\mathop{\sum }\nolimits }_{j=0}^{{N}_{obs}}{\omega }_{obs,j}-\bar {{\omega }_{obs}}}{{N}_{obs}-1} }$
(4)Standard deviation of the step length	$\sqrt{ \frac{{\mathop{\sum }\nolimits }_{j=0}^{{N}_{obs}}{t}_{obs,j}-\bar {{t}_{obs}}}{{N}_{obs}-1} }$

We used the R package “abc” (Csilléry, Franois & Blum (2012), http://cran.r-project.org/web/packages/abc/index.html) to perform the analysis. This package uses a standardized Euclidean distance to compare the observed and simulated summary statistics. We present results for the two regression-based correction methods and for the basic rejection ABC method.

### Simulations

We did two simulation experiments. First we assessed the performance of the three ABC methods for our model. Then, we evaluated how well these methods approximate posterior probabilities depending on the relation between the temporal scales of simulated trajectories and their observations. For both experiments we used a set of one million simulated trajectories, with parameters *κ* (dispersion parameter for the turning angles) and *λ* (parameter for the times between changes of direction) drawn from the priors *p*(*κ*) = *U*[0, 100] and *p*(*λ*) = *U*[0, 50]. The choice of uniform priors was because the aim of our simulation study is to analyze the performance of the ABC methods to fit the model under a diversity of scenarios. Using uniform priors it is possible to consider different values for the parameters with equal probability. The number of simulated steps was such that all the trajectories had at least 1500 observations. All trajectories were observed at regular times of Δt=0 .5. The R code is available in https://github.com/sofiar/ABC-steps-and-turns-.

#### Assessment of the inference capacity of the ABC methods

We assessed the performance of the three ABC versions: simple rejection, rejection corrected via linear regression and rejection corrected via neural network. For different values of threshold (*ϵ*) and for each algorithm version we conducted an ABC cross validations analysis. That is, we selected one trajectory from the million reference set and used it as the real one. We did this selection in a random manner but with the condition that the parameters chosen were not close to the upper limit of the prior distribution. In order to avoid the bias produced by the use of uniform priors in the artificial upper limits of them, we only considered the estimations of *λ* ≤ 25 and *κ* ≤ 70. Then, parameters were estimated using different threshold values (*ϵ*) with the three algorithms and using all simulations except the chosen one. This process was replicated *N*_*rep*_ = 100 times. For each method and *ϵ* value, we recorded the posterior samples obtained for both *λ* and *κ*. We then calculated the prediction error as

(3) \begin{eqnarray*}\sqrt{ \frac{\sum _{i}(\tilde {{\theta }_{i}}-{\theta }_{i})^{2}}{{N}_{rep}} } \theta =(\lambda ,\kappa );i=1,\ldots ,{N}_{rep}\end{eqnarray*}

where *θ*_*i*_ is the true parameter value of the *i*th synthetic data set and ${\tilde {\theta }}_{i}$ is the posterior median of the parameter. We also compute a dispersion measure of the errors in relation to the magnitude of the parameters for each method and tolerance value. We call dispersion index (*DI*) to this quantity and we calculate it as

(4) \begin{eqnarray*}DI= \left[ \sum _{i} \frac{{|}\tilde {{\theta }_{i}}-{\theta }_{i}{|}}{{\theta }_{i}} \right] /{N}_{rep} \theta =(\lambda ,\kappa );i=1,\ldots ,{N}_{rep}\end{eqnarray*}

Furthermore, in order to assess whether the spread of the posterior distributions were not overly large or small, we computed the empirical coverage of the *α* = 95 percent credible interval for the two parameters and for different thresholds (*ϵ*). The empirical coverage is the proportion of simulations for which the true value of the parameter falls within the *α*% highest posterior density interval (HPD). If the nominal confidence levels were accurate, this proportion should have been near 0.95. If this is true for all *α*, it is said that the analysis satisfies the *coverage property*. A way to test this property is by performing the Coverage Test and it is also a useful way to choose the threshold value *ϵ*. This test was first introduced by Prangle et al. (2014) and the basic idea is to perform ABC analyses under many data sets simulated from known parameter values and for each of them compute *p*, the proportion of the estimated posterior distribution smaller than the true parameter. Ideally these values should be distributed as a *U*(0, 1). For a complete description of this test see (Prangle et al., 2014). In order to analyze all possible *α* values, we performed this test using the package “abctools” (Nunes & Prangle (2015); https://cran.r-project.org/web/packages/abctools/index.html).

#### Relative scale of observations and accuracy of the posterior density

**Figure 2:**
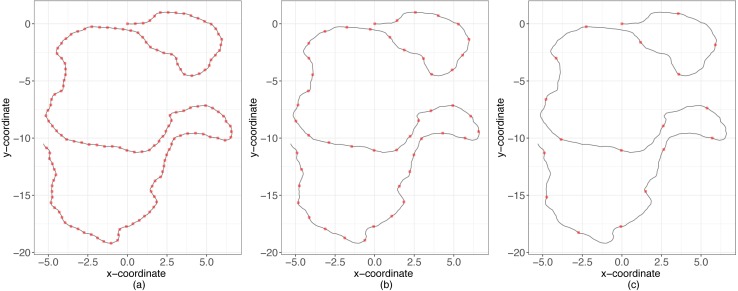
Simulations examples. Sampling schemes and the temporal scale of changes in direction. With black lines the movement process and with red points the observation. (A) Over-sampling case, (B) balanced case, (C) sub-sampling case.

We continued the analysis evaluating how well these methods approximate posterior probabilities as a function of the ratio, *R*, between the temporal scale of observation (Δ*t*) and the temporal scale for changes in directions (1∕*λ*) (Fig. 2). For instance, if *R* = 1 then *λ* = 1∕Δ*t*, which means that the time between consecutive observations is equal to the mean time between changes in direction. Conversely, if *R* < 1 then the time scale between consecutive observations is smaller than the time scale at which animals decide to change directions (over-sampling case), and the opposite occurs if *R* > 1 (sub-sampling case) (Fig. 2). We considered different values of *R* (between 0.06 and 5) and for each simulated 50 trajectories with values of *κ* ∈ {10, 20, 30, …, 70}. Then, using the original million trajectories, the estimations for the three methods of ABC were computed considering these new trajectories as the true observations. We calculated the predictor error for *κ* and *λ* for every combination of *R* and *κ*.

### Sheep data example

In the case that there is only location data available, i.e., GPS data at each time Δ*t*, we have concluded that as it is not possible to know how many changes of directions were between consecutive observations, the likelihood function resulted to be intractable, making ABC techniques useful methods to make inference about the model. Suppose that there exists a source of information that permits estimation of the values of *t*_*i*_ and *ω*_*i*_ ∀*i*. Considering these estimated values as the truth, we have samples *t*_1_, …, *t*_*n*_ and *ω*_1_, …, *ω*_*n*_ and therefore the estimation of *κ* and *λ* is direct.

We compared both estimations for a real trajectory. As we have presented thus far, we fit the model using only information from the GPS locations with ABC techniques. In addition, we used information from DailyDiary devices in order to reconstruct the high-resolution trajectory. This allowed us to infer the times at which the animal changes direction, obtain samples of *t* and *ω* and use MCMC to obtain draws from the posterior distributions of the parameters. The data used were collected from one sheep in Bariloche, Argentina, during February and March of 2019. The sheep was equipped with a collar containing a GPS (CatLog-B, Perthold Engineering, http://www.perthold.de; USA), that was programmed to record location data every five minutes, and a DailyDiary (DD, Wilson, Shepard & Liebsch, 2008), that was programmed to record 40 acceleration data per second (frequency of 40 Hz) and 13 magnetometer data per second (frequency of 13 Hz). The DD are electronic devices that measure acceleration and magnetism in three dimensions, which can be described relative to the body of the animal. Such data allow the Dead-Reckoned (DR) path of an animal to be reconstructed at high resolution. In some cases there is no such data, and only information of the location at certain times is available. The goal here is to compare the ability of the ABC’s methods to estimate the parameters of the model when only information of the location at certain times is available, with the estimation of MCMC techniques when extra information is available.

From the original data, we randomly selected one segment of 6 hours. Using the DD information, we first estimated the path traveled (pseudotrack) by the sheep using the dead-reckoning technique. (Wilson & Wilson, 1988; Wilson et al., 2007). In this step we made use of the R package “TrackReconstruction” (https://cran.r-project.org/web/packages/TrackReconstruction/index.html). After that, we corrected the bias of the estimations using the data from the GPS (Liu et al., 2015). This correction was made using the R package “BayesianAnimalTracker” (https://cran.r-project.org/web/packages/BayesianAnimalTracker/index.html). In this manner, we obtained a trajectory sampled with a resolution of 1 s. To satisfy the hypotheses of the model, we selected part of that trajectory that appeared to come from the same behaviour, i.e., we selected a piece of the trajectory that visually appeared to have the same distribution of turn angles and step lengths.

To determine the points at which there was a change of movement direction, we applied the algorithm proposed by Potts et al. (2018) that detects the turning points of the trajectory using data of the animal headings and subsequently calculated steps and turning angles. With that information it is straightforward to infer the parameter’s values via MCMC techniques and obtain samples from the joint posterior distribution directly using the software Stan as the likelihood can be written in closed form (Carpenter et al., 2017).

We also calculated the summary statistics of the trajectory observed at *dt* = 50 secs (1 observation every 50 of the reconstructed trajectory) and applied the three ABC algorithms. We finally compared both estimations. The data used is available at Figshare with DOI (10.6084/m9.figshare.9971642.v2).

## Results

### Assessment of the inference capacity of the ABC methods

**Figure 3:**
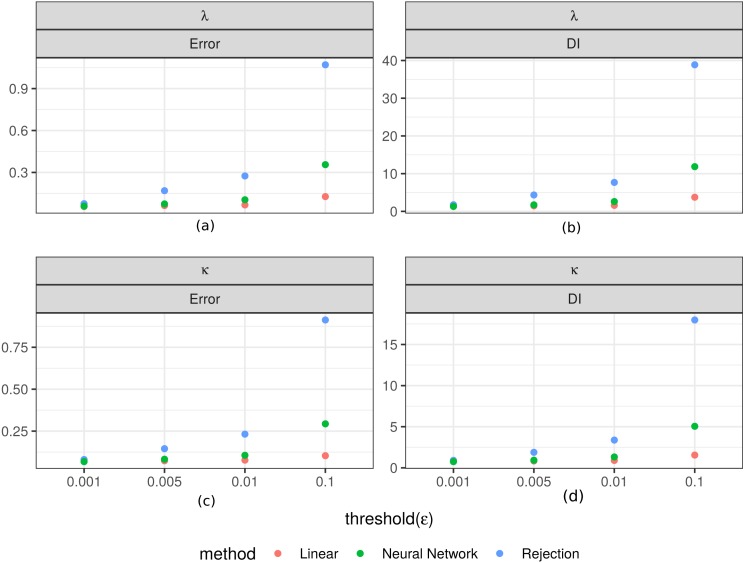
Prediction errors and dispersion index from the ABC cross validation analysis. Values for the prediction errors (Eq. (3)) and for the dispersion index *DI* (Eq. (4)) for: the rate parameter for the duration of steps *λ* (A–B), and the concentration parameter for the turning angle between consecutive steps *κ* (C–D), in each method and threshold *ϵ*.

**Figure 4:**
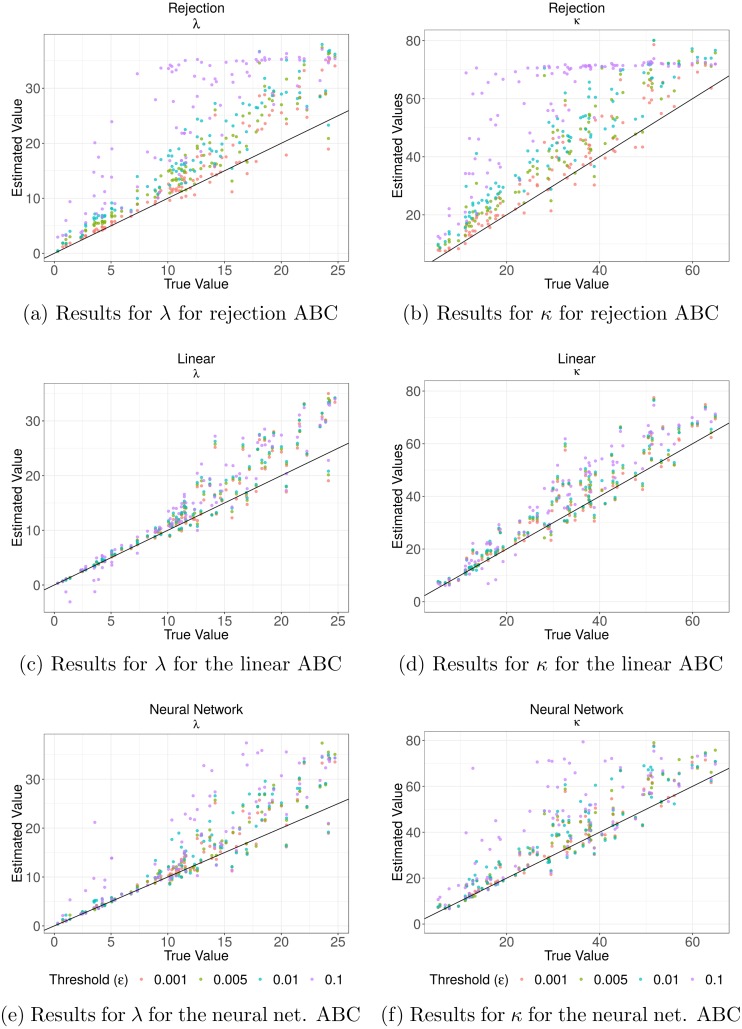
Cross validation analysis for the Rejection ABC algorithm and the two corrections. In (A) and (B) the results for both parameters for the rejection ABC; in (C) and (D) for the linear ABC; and in (E) and (F) for the Neural Network ABC. The relationship between the true parameter values and the median of the estimated posterior are shown. Colors denote the results for different threshold values (*ϵ*): purple for *ϵ* = 0.1, blue for *ϵ* = 0.01, green *ϵ* = 0.005 and red for *ϵ* = 0.001. The black line indicates the line *x* = *y*—the ideal relation.

Figure 3 shows the values of the prediction errors and the dispersion index (*DI*) for each method and *ϵ* value obtained from the ABC cross validation analysis. In all cases the prediction errors decreased when the value of the threshold (*ϵ*) decreased. However, for the algorithms corrected via linear regression and neural networks larger threshold levels (*ϵ*) can produce lower prediction errors. Something similar happened with the *DI* values: lower threshold values imply lower values of this index (Fig. 3). These values give us an idea of the width of the posterior distributions. It is evident that for the case of the rejection algorithm the posterior distributions are quite wide, especially for *ϵ* = 0.1. However, for the corrected algorithms we can assume that the difference between the estimated and true parameters are up to approximately 1.3 units for *κ* and in 0.3 for *λ* in the best case (*ϵ* = 0.001). We also analyzed the relationship between the true parameter values and the median of the estimated posterior (Fig. 4). The estimate of *λ* improves when it takes lower values, especially for the algorithm corrected via linear regression. That behaviour is not so clear for the parameter *κ*. We further elaborate this point in the discussion. Based on these results, the algorithm corrected via linear regression seems to perform the best.

**Figure 5:**
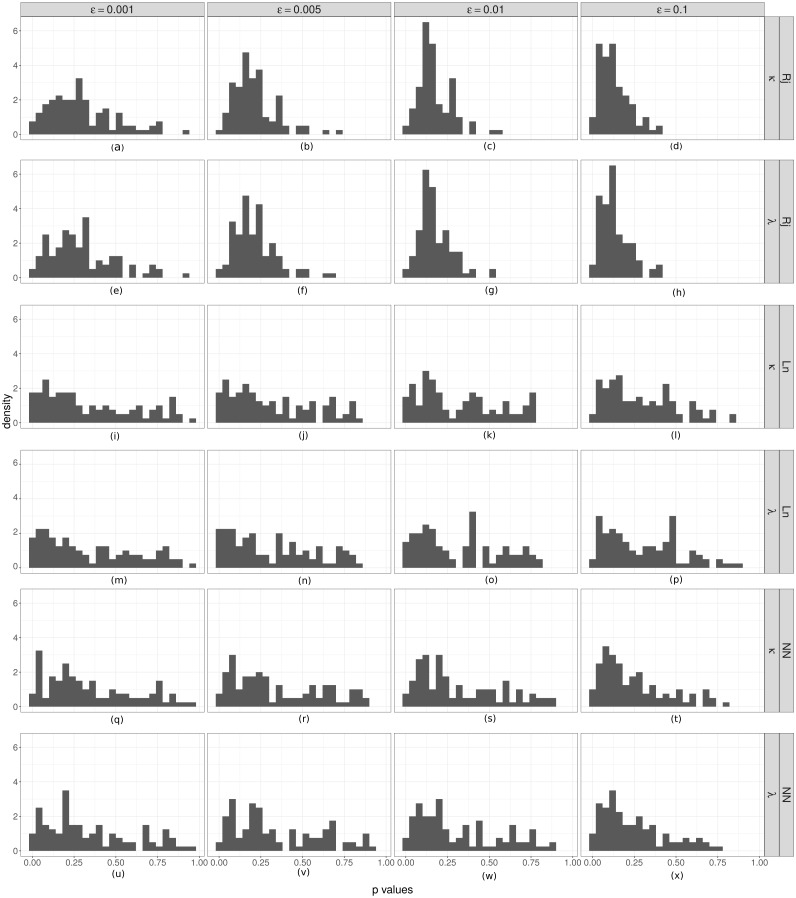
Coverage analyses for parameter estimation. Coverage test for *λ* (rate parameter for the duration of steps) and *κ* (concentration parameter for the turning angle between consecutive steps). Is is shown the relative frequency (*p*) of accepted parameter values that were less than the true value in ABC analyses. If the spread of the posterior distributions are not overly large or small, these values should be distributed as a *U*(0, 1). From (A) to (X): by column the results for different *ϵ* values. The first two rows correspond to the results of the Rejection algorithm, the second two to the ABC with linear correction, and the last two to the ABC corrected via neural networks.

We estimated the empirical coverage of the 95% HPD intervals for both parameters (*κ* and *λ*), for the three ABC algorithms and for different threshold (*ϵ*) values. Almost always, these indices were greater than 95, except in the case of the highest threshold value (*ϵ* = 0.1) for the simple rejection algorithm and the one corrected via neural network, for which the empirical coverages were slightly below 0.95. The plots for this analysis are provided in the Appendix S1.

Finally, in order to check the *coverage property* we performed a coverage test for both parameters (Fig. 5). In most cases, the distributions obtained do not show a clear approximation to a *U*(0, 1). However there is an evident difference between the histograms obtained with the simple rejection ABC and those obtained with the other two algorithms. The shapes of the rejection ABC are those that are farthest from being uniform: for both parameters the distributions of the *p* values are left skewed indicating that the algorithm tends to overestimate the parameters. For the other two algorithms the left skewed is much moderate, and in the case of the lowest *ϵ* values for the linear algorithm the histograms are more uniform, indicating that its *coverage* could be being reached. However, not rejecting that coverage holds does not unequivocally demonstrate that the ABC posterior approximation is accurate. If the empirical data is uninformative, the ABC will return posterior distributions very similar to the priors, which would produce uniform coverage plots.

### Relative scale of observations and accuracy of the posterior density

**Figure 6:**
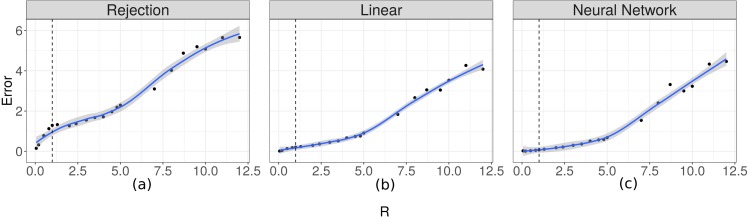
Prediction errors for *λ*. Scatter plot-and-smoother graph (by local regression) of the prediction errors for the rate parameter for the duration of steps (*λ*) for different ratios between the temporal scale of observation and the scale for changes in directions (*R*). In (A) the results for the Rejection ABC, in (B) for the Linear ABC, and in (C) for the Neural Network ABC. High *R* values indicate that the temporal scale of the observation is higher than the temporal scale of the the movement decision process. The black dots are the prediction errors for *λ* for each *R* value. The blue line is the smoothed curve for those values. The 95% intervals are shown in gray. The vertical dotted line indicates *R* = 1.

**Figure 7:**
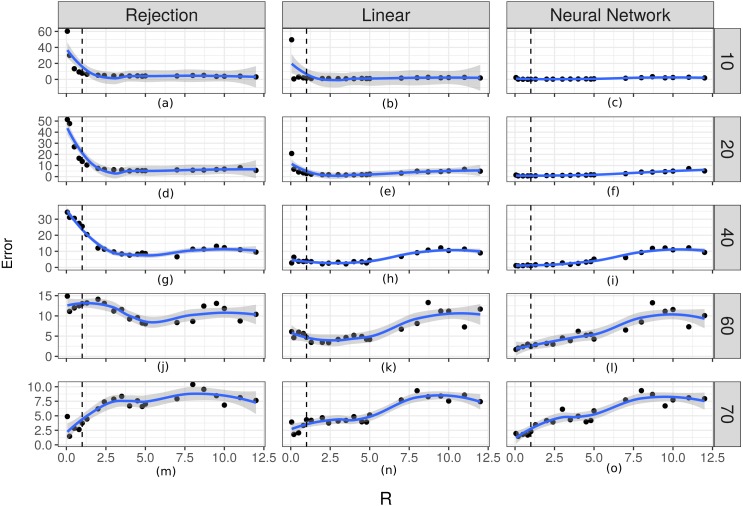
Prediction errors for *κ*. From (A) to (O) the scatters plot-and-smoother graphs (by local regression) of the prediction errors for the dispersion parameter of the turning angles (*κ*) for different ratios between the temporal scale of observation and the scale for changes in directions (*R*). Rows correspond to different values of *κ* and columns to different ABC algorithms. High *R* values indicate that the temporal scale of the observation is larger than the temporal scale of the the movement decision process. Black dots are the prediction errors of *κ* for each *R* value. The blue line is the smoothed curve for those values. The 95% intervals are shown in gray. The vertical dotted line indicates *R* = 1.

In order to evaluate the importance of the relationship between the time scale of the observation and the time at which changes occur in the movement process, we evaluated how well the two parameters fit in relation to the R ratio. The prediction errors for *λ* increased as the value of *R* increased (Fig. 6). For the case of the prediction errors for *κ* this relation can be seen when the true value of this parameter takes larger values (Fig. 7).

For the case of the prediction errors for *κ* this relation can be seen when the true value of this parameter takes larger values. Again, the corrected algorithms have the smallest errors for both parameters.

According to the results shown in Figs. 6 and 7, it is evident that there is a relationship between the ratio *R* and the capacity of these methods to estimate the parameters. For rates approximately less than 5 the errors are small and it is possible to obtain good estimates. This necessitates that the time scale of the observation be approximately less than 5 times the time-scale at which the animals decide to change direction. For higher values of Δ*t* it would be more difficult to make inferences using this technique.

### Sheep data

**Figure 8:**
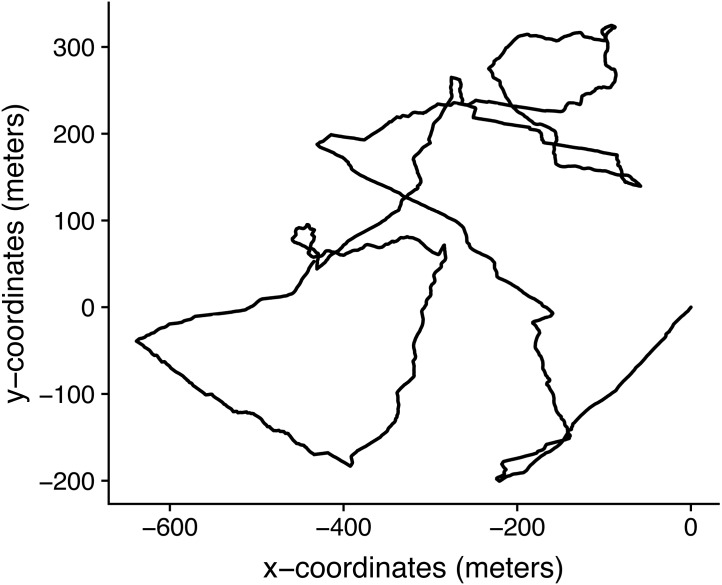
Trajectory from February 27th, 2019 from 19: 01: 21hs to 20: 02: 00hs, reconstructed with one second resolution by dead-reckoning and corrected using the GPS information.

The selected trajectory was from February 27th, 2019 from 19:01:21hs to 20:02:00hs, a total of 1.01 hours (Fig. 8). For the estimations made using the reconstruct high resolution trajectory, the posterior distribution of each parameter was estimated from a sample of 3 × 1000 MCMC draws. As they were in the lower limits of the prior distributions, to conduct inference via ABC with only the location data, we simulate a new set of trajectories with priors of *p*(*κ*) = *U*[0, 10] and *p*(*λ*) = *U*[0, 10].

**Figure 9:**
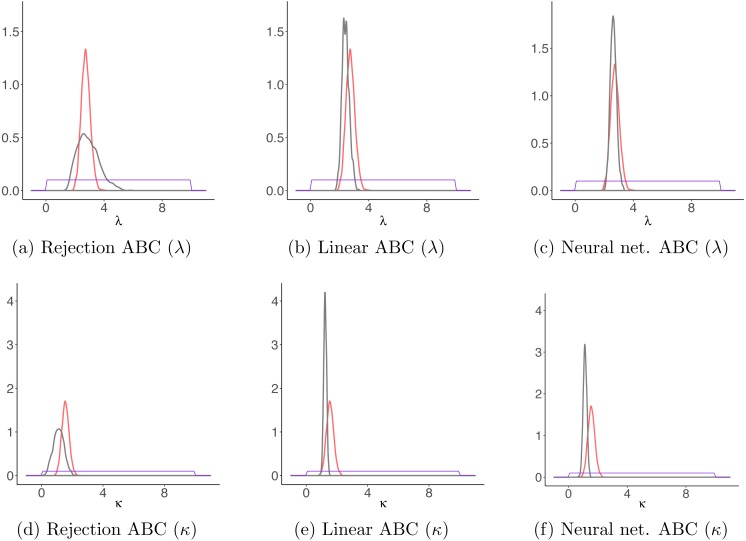
Results for the sheep data example. Comparison between parameter estimates for the sheep movement trajectory based on the detailed trajectory reconstructed using magnetometer and GPS data, and those estimated based on the trajectory sampled at regular time intervals. With red, the posteriors obtained for both parameters *κ* and *λ* through MCMC on the detailed trajectory and with gray those obtained with each ABC algorithm on the observed locations. The purple lines correspond to the priors distributions. (A–C) The results for *λ* for each ABC algorithm; (D–F) the results for *κ* for each ABC algorithm.

Draws from the posterior distributions obtained through MCMC and through the ABC algorithms gave similar results (Fig. 9). Again, the rejection ABC algorithm produced the estimation which is less exact, i.e., the posterior is the furthest from the one obtained by MCMC. Although this trajectory is just a simple example, it shows that it is possible to apply this model to actual animal trajectories.

## Discussion

Animal movement modeling and analysis is either considered in continuous or discrete time. Continuous time models are more realistic but often harder to interpret than the discrete versions (McClintock et al., 2014). A compromise between these approaches is to model movement as steps and turns but to have step duration (or the times at which turns are made) occur in continuous time. In this manner one can have the best of both worlds, so to speak. Here we considered that the underlying movement process, evolving in continuous time, is observed at regular time intervals as would be standard for a terrestrial animal fitted with a GPS collar. The likelihood function resulted to be intractable, but it is feasible to quickly generate simulations from the process and observation models. Thus, we proposed to use ABC methods. Even though these techniques showed certain limitations, it was possible to obtain accurate parameter estimates when the temporal scale of observations was not too coarse compared to the scale of changes in direction.

Our simulation study showed that simple rejection ABC does not perform well for the proposed state-space model but the two corrected versions of this algorithm improve estimations (Figs. 3 and 4). Overall, the best performance was obtained with the linear correction. However, the applicability of these methods depends strongly on the rate between the observation’s scale and the mean time between changes in movement direction. We found that when this ratio is smaller than 5 it is possible to make inferences about the parameters (Figs. 6 and 7). That is, it would be necessary that the observations are less than 5 times the average of the times between changes of directions in order to be able to generate good estimations.

Beyond our findings about the capacity to make inference with these techniques in a simulation study, it is important to note that in an applied case more informative priors could be considered. Here, our aim was to evaluate the performance of the ABC techniques considering several parameter combinations generating trajectories and then sampling from those trajectories. In order to optimize computing time, we simulated a million trajectories sampling their parameters from uniform distributions and then we randomly choose one of them as observed data while the rest of the simulations was used to perform the ABC computations. That justify the use of uniform priors for our parameters. As we did in our real data example, in applied cases it wold be relatively straightforward to come up with more informative priors, especially for the expected time for changes in movement direction. As new technologies allow us to obtain very detailed movement data, we can have better estimates of the temporal scales at which animals make movement decisions. As we did in our real data example, high-frequency data from accelerometers and magnetometers combined with GPS data can be used to obtain trajectories with sub-second temporal resolution to then detect fine-scale movement decisions such as changes in direction. These detailed trajectories could be used to elicit informative priors to use when only location data at longer time frequencies is available.

## Conclusions

The movement model presented here is quite simple as we assume constant movement speed and turning angles with zero mean. Nevertheless, the model is an improvement over discrete-time versions where the temporal scale of movement has to match the scale of observations. Further developments of these methods should be considered, for example adding a stochastic error term in the observations, or allowing for the presence of missing values in them. It should also be important to contemplate additional features that are common in movement studies such as the inclusion of more than one movement behavior and the effect of habitat features on both movement parameters and changes among behaviors (Morales et al., 2004; Hooten et al., 2017). Even though these extensions would mean estimating several parameters, such models will imply further structure in the trajectories that could be used as part of the summary statistics used to characterize the data. Hence, it might reduce the combination of parameters values capable of reproducing features present in the observations, allowing for ABC inference.

In general, the processes behind the realized movement of an individual and the processes that affect how we record the trajectory are usually operating at different time scales, making it challenging to analyze and to understand the former given the latter. The state-space model used here allowed us to connect these two scales in an intuitive and easy to interpret way. Our results indicate that, when designing data collection protocols, it is crucial to be aware of the differences between the time scale in which animals make their movement decisions and the time scale at which the data will be collected, in order to avoid incorrect interpretations of the system. In addition, very fine time scales may not be necessary to have good estimates of certain movement processes.

##  Supplemental Information

10.7717/peerj.8452/supp-1 Appendix S1 Supporting Information

## Additional Information and Declarations

### Data Availability

The following information was supplied regarding data availability: The raw data is available at Figshare: Ruiz Suarez, Sofia; Morales, Juan Manuel (2019): GPS and DD sheep data. figshare. Dataset 10.6084/m9.figshare.9971642.v3. R codes are available at Github: https://github.com/sofiar/ABC-steps-and-turns-.
