# Supplementary material for: Using approximate Bayesian inference for a “steps and turns” continuous-time random walk observed at regular time intervals
Source: PeerJ. 2020 Feb 11;8:e8452. doi: 10.7717/peerj.8452 (PMC7020826; doi:10.7717/peerj.8452)
Supplement: Appendix S1 [file peerj-08-8452-s001.pdf]

Supporting Information

1 Calculation of the complete data likelihood

Lets consider the variable $M_i = (\mu_{i,1}, \mu_{i,2})$ for describing the position (in $x-y$ coordinates) of the latent process by step i and the variable $O_j = (o_{j,1}, o_{j,2})$ for the position of the observation j. Lets remember that we defined N_j as the amount of steps (or changes of direction) that the animal took from time 1 to time $j(\Delta t)$.

We have that $\mu_{0,1} = 0$ and $\mu_{0,2} = 0$

For $i = 1, ..., N_{steps}$

$$\mu_{i,1} = \mu_{i-1,1} + cos(\phi_{i-1})t_{i-1}$$
$$\mu_{i,2} = \mu_{i-1,2} + sin(\phi_{i-1})t_{i-1}$$

And then it is possible to parameterize the observation process as

$o_{0,1} = 0$ and $o_{0,2} = 0$ and for $j = 1, ..., N_{obs}$

$$o_{j,1} = \mu_{N_j,1} + cos(\phi_{N_j})\left(j\Delta t - \sum_{k<N_j-1} t_k\right)$$
$$o_{j,2} = \mu_{N_j,2} + sin(\phi_{N_j})\left(j\Delta t - \sum_{k<N_j-1} t_k\right)$$

So o_j is a function of all positions M_i from $i = 0$ to $i = N_j$. Then $O_j = h(M_{0:N_j})$, where $M_{0:D} = (M_0, M_1, M_2, ..., M_D)$. Lets suppose that we know the number of changes of direction that the animal took between consecutive observations, so we know the $N_j \forall j$. Then the likelihood of the SSM with different temporal scales for an individual trajectory is given as

$$L(\kappa, \lambda, M, O) = P\left(O_0 = o_0, O_1 = o_1, \cdots, O_{N_{obs}} = o_{N_{obs}}\right)$$
$$= P\left(h(M_{0:N_1}) = o_1, h(M_{0:N_2}) = o_2, ..., h(M_{0:N_{N_{obs}}}) = o_{N_{obs}}\right)$$

As $O_j = h(M_{0:N_j})$, in order to get a formulation of L it is necessary to obtain the distributions of M_i (1) and for $O_j = h(M_{0:N_j})$ (2).

Step 1: Formulation of (1)

We are looking for a formulation for $M_i = (\mu_{i,1}, \mu_{i,2})$. We are going to consider just the variable corresponding to the x-coordinate ($\mu_{i,1}$), the second is analogous.

We have that

$$\mu_{i,1} = \mu_{i-1,1} + cos(\phi_{i-1})t_{i-1}$$

with $\phi_i \sim vonMises(\phi_{i-1}, \kappa)$ and $t_i \sim Exp(\lambda)$. To obtain the distribution of $\mu_i|\mu_{i-1}$ it is necessary to obtain the distribution form of $Z = cos(\phi)t$. Using the Change of variable Theorem it is possible to calculate this distribution. To do that, lets first consider $V = g(\phi) = cos(\phi)$. We want to obtain an expression for f_V. Splitting the domain of g and applying the Transformation Method Theorem, is obtain:

$$f_V = (f_\phi(-acos(v)) + f_\phi(acos(v)))\frac{1}{\sqrt{1-v^2}}I_{-1\leq v\leq 1}(v)$$

Now we can calculate f_Z as $f_Z = Vt$. Again making use of the Transformation Method Theorem and using the fact that the times and angles are independent, it is possible to obtain the following expression

$$f_Z(z_1) = \int f_V(\frac{z_1}{z_2})f_t(z_2)I_{\{-z_2\leq z_1\leq z_2\}}(z_1)I_{\{z_2>0\}}(z_2)\cdot dz_2$$

Having f_Z obtaining $p(\mu_i|\mu_{i-1})$ is immediate.

Step 2: Formulation of (2)

Now, we are looking for a formulation for $O_j = h(M_{0:N_j})$. Les write

$$O_j = (o_{j,1}, o_{j,2}) = \big(h_1(\mu_{0:N_j,1}), h_2(\mu_{0:N_j,2})\big)$$

Again, we are going to consider just the variable corresponding to the x-coordinate ($o_{j,1}$), the second is analogous.

We have that

$$\begin{aligned} o_{j,1} &= h(\mu_{0:N_j,1}) \\ &= \mu_{N_j,1} + \cos(\phi_{N_j}) \left(j\Delta t - \sum_{k<N_j-1} t_k \right) \\ &= \mu_{N_j,1} + V_{N_j}(c_j - W_{N_j-1}) \end{aligned}$$

We already know the distribution of V_{N_j}. The distribution of W_{N_j-1} is just a sum of $N_j - 2$ $Exp(\lambda)$, a $\Gamma(N_j - 2, \lambda)$. If we consider $\tilde{W}_{N_j-1} = c_j - W_{N_j-1}$ (which differs with W_{N_j-1} just in a constant), we have that $f_{\tilde{W}_{N_j-1}}(v) = f_{W_{N_j-1}}(c_j - v)$

So, we can rewrite $o_{j,1}$ as

$$o_{j,1} = \mu_{N_j,1} + (V_{N_j})(\tilde{W}_{N_j-1}) = \mu_{N_j,1} + S_{N_j-1}$$

To obtain the distribution of S_{N_j-1}, again is necessary to use the Transformation Method Theorem and the independence between the times and the angles:

$$f_{S_{N_j-1}}(s_1) = \int f_{V_{N_j}}(\frac{s_1}{s_2}) f_{\tilde{W}_{N_j-1}}(s_2) I_{\{-s_2 \le s_1 \le w_2\}}(s_1) I_{\{s_2 \le -c_j\}}(s_2) \cdot ds_2$$

2 Summary Statistics

We provide the plots of the summary statistic analyzed versus the parameters (Figures 1, 2 and 3). We choose four that attempt to describe the trajectories in an integral way and characterize them according to parameter values. The selected were the ones of Figure 1(a-d).

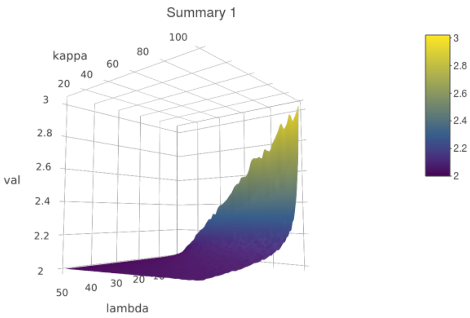

(a) Point estimate for λ

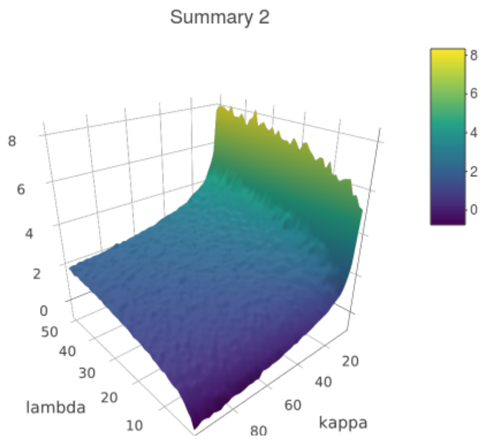

(b) Point estimate for κ

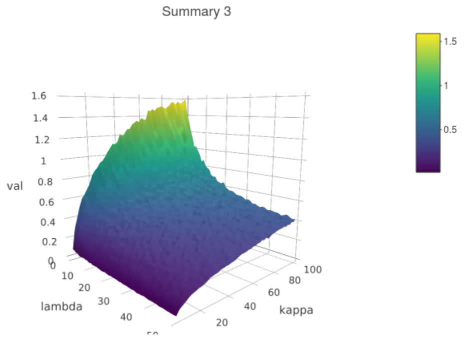

(c) Standard deviation of the turning angle

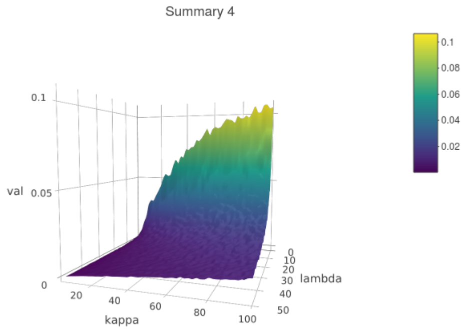

(d) Standard deviation of the step length

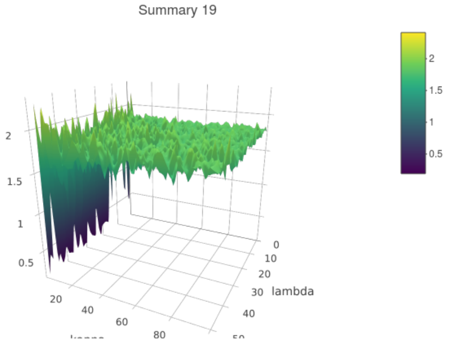

(e) Standard deviation of the directions

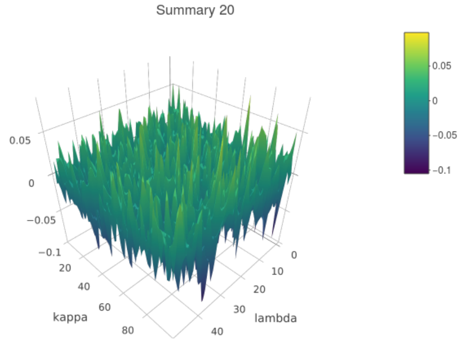

(f) Third lag of the autocorrelation function of the turning angles

Figure 1: Plots of the summary statistics vs the simulated parameters

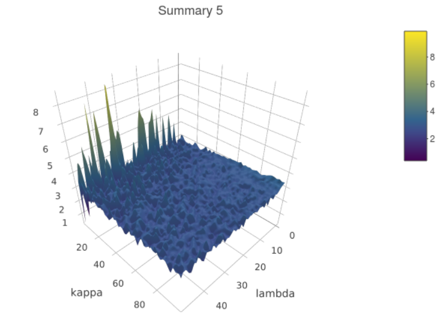

(a) Sinuosity index

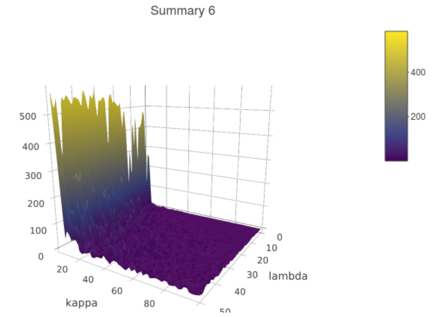

(b) Mean square displacement

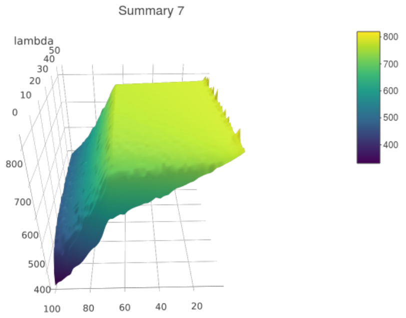

(c) Length of the trajectory

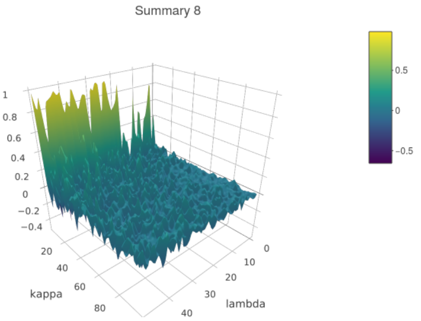

(d) Mean of the cosine of the turning angle

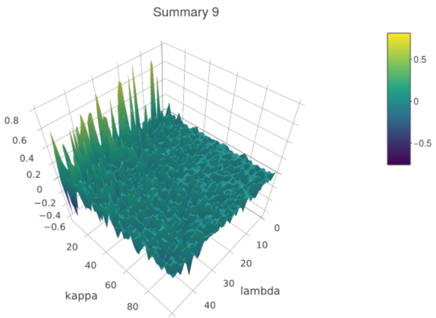

(e) Mean of the sine of the turning angle

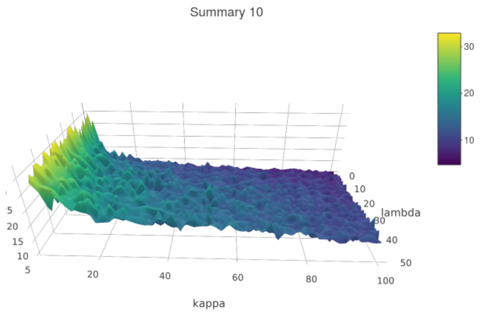

(f) $\sqrt{|(y_{.,1_{max}} - y_{.,1_{min}}| + |y_{.,2_{max}} - y_{.,2_{min}}|}$

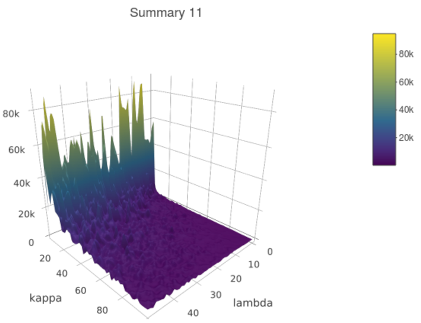

(g) Area enclosed by the trajectory

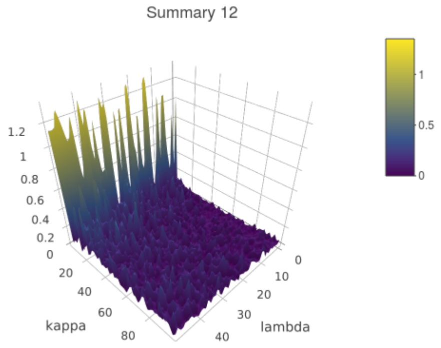

(h) Tortuosity index

Figure 2: Plots of the summary statistics vs the simulated parameters

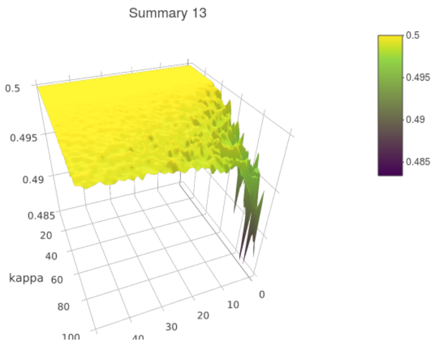

(a) Maximum step length

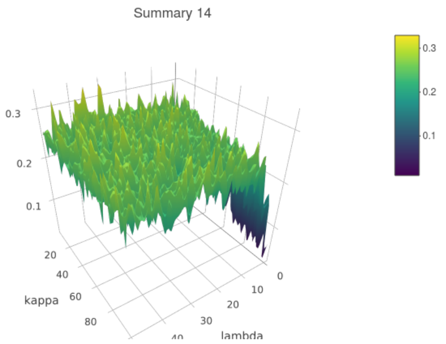

(b) Second lag of the autocorrelation function of the turning angles

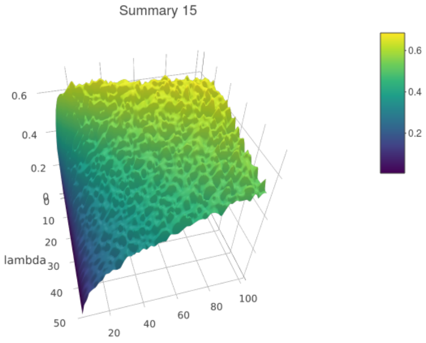

(c) Mean of the areas enclosed by the path every 5 observations

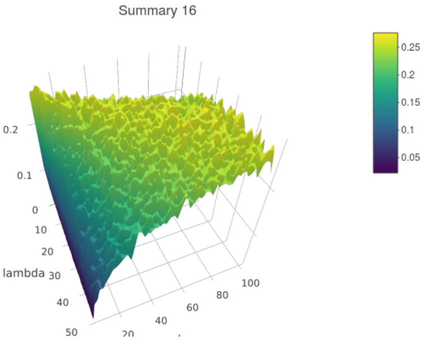

(d) Standard deviation of the areas enclosed by the path every 5 observations

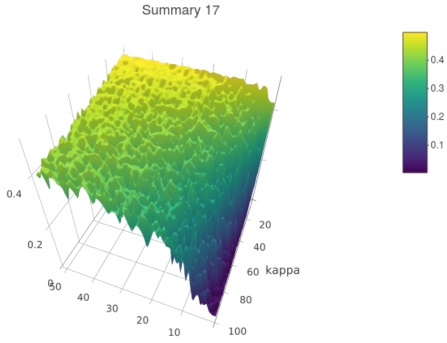

(e) Minimum step length

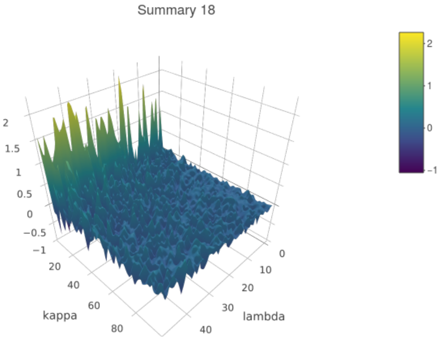

(f) Mean direction

Figure 3: Plots of the summary statistics vs the simulated parameters

3 Empirical Coverage

We present the results for the empirical coverage of the 95% high posterior density(HPD) intervals for the two parameters. This value is the proportion of simulations for which the true parameter value falls within the 95% HPD interval. If the posterior distributions were correctly estimated, this proportion should have been near 0.95. We compute this index for both parameters (κ and λ) and for the three ABC algorithms: Simple Rejection, Corrected via Linear Regression and Corrected via Neural Network. We did that for threshold (ϵ) values of: 0.001, 0.005, 0.01 and 0.1.

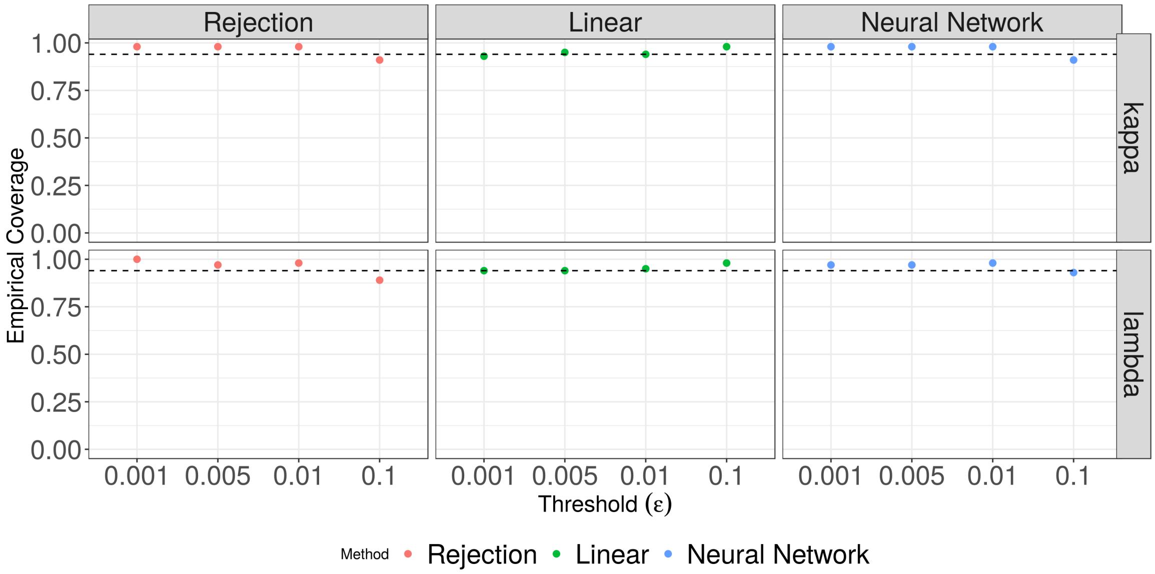

Figure 4: Empirical Coverage obtained for κ and λ for different threshold values. By row the results for every parameter and by column the results for the three ABC algorithms. The dash line indicates the value 0.95
